# A Chat about Health

**Published:** 1858-05

**Authors:** M. Gowran

**Affiliations:** Griswold’s Mills, N. J.


### A CHAT ABOUT HEALTH.

By M. Gowran.

If you will lend us your attention, reader, we will relate a few things concerning health. *First*, Health sustains an important interest in social life. If we meet or hear from a friend, the first thought or inquiry is about health. It is the only *sure* foundation for beauty; and *that* by many is considered a priceless treasure, and coveted by the mass of mankind. Yet how many American ladies will keep within doors for weeks together, for fear the summer sun will brown their lily face; when if they would ramble on the hill-top, in the forest glade, and cull the modest wild flowers, &c., their cheeks might reflect the roseate hue of the spring " Arbutus," without " rouge."

Health has a controlling influence over immorality; and also advances the pecuniary interest of mankind. For what can be accomplished without it? Comparatively nothing. It ought to be more highly prized than fame or riches. But alas! how many use it as though it could be bought or sold at their bidding. A kind of employment that brings all the muscles into action daily, is conducive to health. Sedentary habits, reading, writing, sewing, after the hours have walked far into the night, is detrimental to health.

"Consumption, "Throat-Ail," "Dyspepsia," &c., are encroaching, and poisoning the life-current of the American people; and ought we not to become alarmed at the depredations they are making? If we will continue to violate the physical law, we must expect that disease will be the result of our folly. But many attribute their ailments to kind Nature! To such we therefore say: *You are mistaken.* Nature labors for health. Just look at the pearly dew-drop which she gathers to revive the drooping flower. Still, how oft we reject her as a guide; and for a trifling ailment eat boxes of pills, drink bottles of Mr. Somebody's " Compound Syrup," said to cure almost every disease; but alas! it seldom, if ever, eradicates any. Hence, we lose our money, time, and health. Pardon us, dear reader, for speaking so plainly, for our remarks are deduced from experience, not from theory.

*Griswold's Mills, N. J.*

